# Views, experiences and contributory factors related to medication errors associated with direct oral anticoagulants: a qualitative study with physicians and nurses

**DOI:** 10.1007/s11096-022-01448-x

**Published:** 2022-06-22

**Authors:** Abdulrhman Al Rowily, Nouf Aloudah, Zahraa Jalal, Mohammed H. Abutaleb, Vibhu Paudyal

**Affiliations:** 1 School of Pharmacy, Institute of Clinical Sciences, College of Medical and Dental Sciences, Sir Robert Aitken Institute for Medical Research, University of Birmingham, Birmingham, B15 2TT UK; 2 Pharmaceutical Care Department, King Fahad Military Medical Complex (KFMMC), Medical Department, Ministry of Defense, Dhahran, Saudi Arabia; 3 Department of Clinical Pharmacy, College of Pharmacy, King Saud University, Riyadh, Saudi Arabia; 4 Pharmaceutical Care Department, King Fahad Central Hospital, Jazan Health Affairs, Ministry of Health, Jazan, Saudi Arabia

**Keywords:** Direct oral anticoagulants, DOACs, Healthcare professionals, Medication errors

## Abstract

**Background:**

Direct oral anticoagulants (DOACs) have become preferable for the management of thromboembolic events. Recent publications have however identified high volume of medication errors related to DOACs. There is limited literature on why and how such errors occur or happen in clinical practice.

**Aim:**

This study aimed to explore views, experiences, contributory factors related to DOACs medication errors from the perspectives of healthcare professionals.

**Method:**

Semi-structured interviews using online videoconferencing were conducted with physicians and nurses from tertiary care hospitals in three different regions in Saudi Arabia. Questions included views, experiences and perceived factors contributing to errors. Interviews were transcribed verbatim and were thematically analyzed using MAXQDA Analytics Pro 2020 (VERBI Software).

**Results:**

The semi-structured interviews (n = 34) included physicians (n = 20) and nurses (n = 14) until data saturation was achieved. The analysis identified five themes: Factors related to healthcare professionals (e.g. knowledge, confidence and access to guidelines); Factors related to patients (e.g. comorbidity, polypharmacy, medication review, and communication barriers); Factors related to organization (e.g. guidelines, safety culture and incidents reporting system); Factors related to the DOACs medications (e.g. lack of availability of antidotes and dosing issues); and Strategies for error prevention/mitigation (e.g. the need for professional training and routine medication review).

**Conclusion:**

Healthcare professionals identified errors in relation to DOACs as multifactorial including their own and patient lack of knowledge, lack of clinical guidelines and organizational factors including safety culture. Medication review and reconciliation on discharge were key strategies suggested to reduce DOACs related errors. These strategies support the role of pharmacists as direct patients care providers to minimize DOACs errors.

**Supplementary Information:**

The online version contains supplementary material available at 10.1007/s11096-022-01448-x.

## Impact statements

- Identifying factors related to errors associated with DOACs use is very important to maximize their effectiveness and minimise their adverse events.
- Healthcare professionals and patient knowledge and understanding of DOACs are essential to minimize DOACs medication errors.
- It is important to ensure availability of DOACs guidelines and protocols to promote safe prescribing practices.
- Healthcare professional and patient education, medication review and reconciliation on discharge are key strategies suggested to reduce DOACs related errors. These roles empower pharmacists’ role as direct patientcare provider.

## Introduction

Anticoagulants are first line therapeutic and/or prophylactic regimens for thromboembolic events such as deep venous thrombosis (DVT) and pulmonary embolism (PE) [1]. Their indication also includes prevention of stroke in atrial fibrillation (AF) patients [2]. In addition, patients who cannot tolerate conventional warfarin therapy are usually prescribed direct oral anticoagulants (DOACs)among many other uses. DOACs are also commonly known as novel oral anticoagulants and sometimes called Non-Vitamin-K-antagonist Oral Anticoagulants (NOACs) [3, 4]. DOACs are orally administered in fixed doses with no need for prothrombin time (PT) or international normalized ratio (INR) monitoring. Such practical advantages including less food and drug interactions have led to their increased utilization in clinical settings [5]. Their preferential use extends to vulnerable populations including elderly[6], chronic kidney diseases [7] and in morbidly obese patients [8].

A recent systematic review and meta-analysis of 32 studies showed approximately 1 in 5 patients prescribed DOACs experience a prescribing error [pooled prevalence of errors 20% (95% CI 15–25%; I2 = 96%; 95% PrI 4–43%) [9]. Common prescribing errors included over or under dosing, contraindications, missed doses and duplicate therapy [9]. However, contributory factors were rarely reported in the included studies and there was a lack of use of theory to identify contributing factors [9]. Approaching medication errors using theories and models is important for efficient identification, development and implementation of interventions [10].

A previous study conducted in Saudi Arabia using error reports submitted to pharmacovigilance datasets revealed that prescribing error was the most common error type representing 81.4% of all errors [11]. Increased patient age, concurrent comorbidity and polypharmacy, indication and duration for anticoagulation were related to higher incidence of errors [11, 12]. However, there is limited literature on why and how such errors occur or happen in clinical practice.

## Aim

This study aimed to explore views, experiences and contributory factors related to DOACs medication errors from the perspectives of healthcare professionals in Saudi Arabia.

### Ethics approval

The researchers obtained ethical approval from University of Birmingham Research Ethics Committee (ERN_20-0551) on 6/11/2020. In addition, three ethical approvals from all of the three participating hospitals: National Guard Hospital in Riyadh (SP20/212/R), King Fahad Military Medical Complex in Dhahran (AFHER-IRB-2020–015) and King Fahad Central Hospital in Jazan (164/2020) were obtained on 3/06/2021. All participants signed an informed consent before enrollment in the study.

## Method

### Study design

This study used a qualitative design through in-depth interviews. The Consolidated criteria for reporting qualitative research (COREQ) was followed in the reporting of this study.

### Participants and setting

Semi-structured interviews were conducted with HCPs from three different regions in Saudi Arabia: Riyadh, Dhahran and Jazan. One tertiary hospital in each of the three regions were chosen. Hospitals were selected based on the presence of a cardiology department with at least 2 consultant cardiologists. Purposive sampling was undertaken for the recruitment of participants. Participant inclusion criteria were physicians and nurses who had experiences with DOACs prescribing and treatment.

### Data collection

An invitation e-mail was sent to the hospitals to recruit participants into the study. In each hospital, a local collaborator sent the invitation to eligible healthcare professionals. In addition, the collaborators also further explained the study objectives to the participants during face-to-face meetings before obtaining their consent to enroll in the study. All participants were provided with participants’ information sheet (PIS) and consent forms were distributed to be read and signed by the participants before conducting the interviews.

A topic guide including all questions to ask the interviewees (electronic supplementary 1) was also designed to help interviewers be consistent with all participants in the different centers. Literature review conducted by the study authors including systematic review and meta-analysis and primary research around the extent of DOACs errors was helpful in developing the abovementioned guide [9, 13] and in identifying Reason’s Accident Causation Model theory [14]. Reason’s model categorizes human failure in three pathways: active failures (or person approach) and latent failures (or system approach) and error provoking conditions. Active failures are known to have immediate adverse outcomes compared to latent failures. Questions used in the interviews were mainly focusing on the experience in dealing with DOACs, concerns raised during their utilization in their institution, the level of knowledge they have about these drugs, the perceived safety culture regarding their use, in addition to having guidelines and policies in place to maximize DOACs effectiveness and to reduce their errors. Moreover, additional questions about the factors associated with errors happening with these drugs and the participants’ views regarding prevention of such errors. The questions were revised by the study team who included experts in qualitative research and research collaborators in study sites. The topic guide was further reviewed by 5 physicians and 5 nurses. The interviews were conducted in English (as the common language used in the hospitals and many of the HCPs are English speakers), recorded and transcribed verbatim by a professional transcriber.

The semi-structured interviews were undertaken virtually using Zoom videoconferencing as a platform due to limitations caused by the COVID pandemic. The interview took place from September to end of November 2021 and lasted between 40 to 60 min in duration.

### Data analysis

Interviews were thematically analyzed using MAXQDA Analytics Pro 2020 (VERBI Software) [15]. Each transcript was independently analyzed by two authors (AA, NA), a third author checked both versions (VP) for disagreement. Disagreements were resolved by discussions. As the semi-structured interviews progressed, data were analyzed after each interview to develop initial codes and to identify important and new emerging information.

To improve rigor and trustworthiness, each interview was ended by a summary to be validated by the participants and to check any ambiguity. After each interview the two researchers (AA and NA) met and reflected on the information received. Memos (such as reporting interviewee’s facial expressions or hesitancy to answer certain questions adequately) and journaling were recorded during the interviews and utilized throughout data collection and analysis using MAXQDA memos. Data collection continued until interviewers’ noticed saturation (no more new data revealed by the interviewees) in each group of participant HCPs. Data from interviews were kept secure.

## Results

The semi-structured interviews (n = 34) included physicians (n = 20) and nurses (n = 14). Participant's characteristics are presented in Table 1.

**Table 1:** Participants’ characteristics (n = 34)

Participants characteristics	Physicians (n = 20)	Nurses (n = 14)
**Gender**		
Female	2	13
Male	18	1
**Training background years of experience**		
< 5	5	6
05–10	12	5
11–15	0	2
16–20	1	0
> 20	2	1
**DOACs experience (in years)**	**Prescribing**	**Administer**
< 5	12	3
05–10	6	1
11–15	0	8
16–20	1	0
> 20	1	2
**Specialty or department**		
Cardiology	10	2
Internal medicine	9	1
Unspecialized	0	5
Others*	1	6
**Current job title**		
Consultant	5	
Registrar	10	
Resident	5	
Nursing		9
Manager of department		5

*Others include any specialist for physicians, and for nursing practitioners in medical wards or medical isolation wards, respiratory department, and critical care unit

Saturation of the data collection was achieved after 32 interviews with HCPs and further two interviews were conducted to confirm it.

**Fig. 1:**
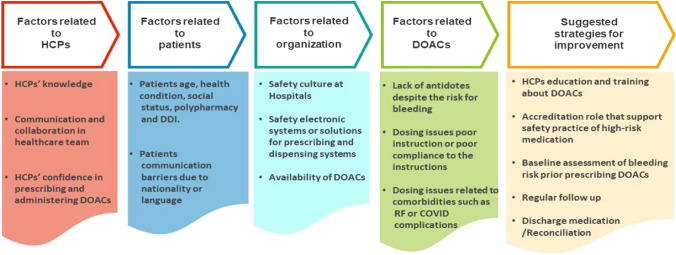
An overview of themes and subthemes emerged during data analysis. HCPs: Healthcare professionals, DOACs: Direct oral anticoagulants, DDI: Drug-Drug interaction, RF: Renal failure, COVID: corona virus infection disease

The analysis of the interviews identified factors associated with medication errors that were categorized under five themes: (1) Factors related to healthcare professionals (HCPs); (2) Factors related to patients; (3) Factors related to organization; (4) Factors related to the DOACs as medications; and (5) Strategies for improvement (Fig. 1).

The following sections describe each theme accordingly with the aid of illustrative quotations.

### Factors related to healthcare professionals

Four sub-themes were identified under factors related to HCPs: HCPs’ knowledge, Communication and collaboration in the healthcare team, and HCPs’ confidence in prescribing and administering DOACs. Below is a description of each subtheme and the supportive quotations.

#### Knowledge of *HCPs*

The participants’ reported knowledge gaps as factors that contribute to the DOACs medication errors. The physicians pointed out that lack of knowledge about the dose, adverse effects, and contraindications were mostly causing such errors:

“..the challenge ……, do you prescribe accordingly, do you adjust the dose? do you overshoot? do you undershoot, do you undertreat duration?..and I think that something that needs to be done, …” Participant 7, physician (Riyadh)

In addition, nurses added that their limited knowledge regarding the medications and their reliance on physicians for education.

“We don't have the knowledge … so, first; the physicians will educate us about this type of medication when they start to give…” Participant 10, Nurse (Jazan)

When referring to clinical guidelines, some participants reported not having access to any structured guidelines or protocols.

“No, … we don't have a written guideline protocol. We had a meeting, discussion but nothing written.” Participant 4, physician (Dhahran)

#### Communication and collaboration amongst *HCPs*

The participants mentioned that good communication among the multidisciplinary team was important to improve DOACs safety.

“…when you prescribe medication, you will discuss first with your team, …… So, the team dynamic and the team in our hospital affect the safety of medication very nicely, in a good way.” Participant 6, physician (Dhahran)

Some participants mentioned that often they did not agree on DOACs choice selection or the dosage and were concerned about the risk but were hesitant to speak up their concerns to the prescribing physician. And when they did, they were not always able to convince the prescribing physician:

“…sometimes worried when It was newly started prescribe DOACs because the risk of bleeding and I have spoken with other colleagues by phone to discuss this case and he was not convinced.” Participant 3, physician (Dhahran)

Nurses felt more comfortable to speak with the consultants:

“…. the thing is they are listening to each other [consultants], except for the residents who will not listen … if I have a problem I will talk to the consultant because they are listening to us.” Participant 9, Nurse (Riyadh)

Physicians and nurses advocated the role of clinical pharmacists especially in multidisciplinary ward's rounds in preventing medication errors:

“Actually, I found like having a clinical pharmacist in our unit is very very very helpful. To be honest with you, I always depend on them on reviewing the medications of the patients and the interactions” Participant 1, physician (Riyadh)

#### *HCPs* confidence in prescribing and administering DOACs

Some HCPs reported confidence in prescribing and administering DOACs. Participants outlined the importance of careful history taking prior to prescribing of these medications is important to ensure their safe use, as this physician describes.

“Anticoagulation are not like any, like, for example, antibiotic or, yeah, anticoagulation I have really [to] concentrate. I have to review all the medication of the patient. I have to go back again to the patient, ask him about the past history of, and if he has any bleeding before; so, it's not like any medication, anticoagulation, yes, yeah.” Participant 1, physician (Jazan)

The importance of having accurate diagnosis at the time of prescribing was also needed for prescriber confidence.

“The physicians in general are becoming more confident in using DOACs when they are clear diagnosis and confirmed clear diagnosis with a thrombosis team” Participant 5, physician (Dhahran)

Physicians’ adherence to standardized prescribing protocols (or systems) and availability of a clinical pharmacist to check before the prescription reached patients were key to improving prescriber confidence and reducing errors:

“Yeah, the good thing in our hospital that they use a well-structured prescribing medication system for the physician to prescribed any medication, it should be approved by pharmacist first and to be reviewed very well regarding if the patient has any side effect or. contraindication to use this agent or contraindication to use this does, the error will be anywhere but it is very uncommon we have in our hospital?” Participant 5, physician (Riyadh)

### Factors related to patients

Participants reported that the health condition and age of the patient affected how patients understood the information given to them.

“We took in consideration the risk factors and the comorbidities of the patient that effect understanding the instruction how to use apixaban the age of patient special the elderly and the cognitive disorder such as Alzheimer disease as well.” Participant 2, physician (Riyadh)

Some participants expressed that patients’ socio-economic status and acceptability of the patient for convincing them also affected the safety of the use of DOACs:

“Some patients are refusing the new medication or even they are receiving it, they are not convinced that they should take. So, I think social factor is one of the most important.” Participant 5, physician (Dhahran)

Some physician participants expressed that some of the errors related to DOACs can be attributed to poor patient understanding of the dosing instructions of these medications, or their poor adherence to the instructions.

“…. patient will take by mistake double the dose, he will, he will take half the dose, he will take it three times instead of one time. …. the problem will come with non-compliance or, the prescriber error in dosing.” Participant 3, physician (Dhahran)

Further HCP participants mentioned that they do assess patients’ interest and ability to understand DOACs before prescribing it.

Effective communication with patients was deemed key to promoting DOACs safety. However, some of the interviewed nurses were non-Arab nationalities and mentioned that they often face language barriers that might contribute to higher errors with DOACs:

“…I believe that one problem that could cause certain errors is, number one, is the language because, sometimes, we're finding it hard, difficult to educate or to inform our patient, especially the elderly… almost all of our patient, we don’t have any sitters available at the bedside to help us communicate with the elderly. so, I think that can cause us problems.” Participant 8, Nurse (Riyadh)

Some participants attributed errors related to the use of DOACs to poly-pharmacy or inadequate medications reconciliation while receiving healthcare at different healthcare facilities:

“The most factor is that sometimes the patient may come to a different clinic and to my clinic, and then ask for medication, and then he want to have a refill for the whole…. and then he'll have big stock, you know, and I think, this way, the patient might be mistakenly taking the dose in the wrong way, you know” Participant 4, physician (Jazan)

Some physicians reported that the safest approach is the one that involved the patient, the family and the pharmacist while prescribing DOACs in a shared decision-making process:

“Our system is very nice and organized…, First of all before we start … we discuss with the family or the patient himself, we explain for them the side effects and the dose and duration and everything, if we are not sure about the dose.. we have our clinical pharmacist to guide us for the dose…” Participant 2, physician (Riyadh)

### Organizational factors

Participants discussed the importance of organization's patient safety culture and electronic prescribing systems on DOACs medication errors. Although most of the participants expressed feeling safe due to the quality and nature of safety measures adopted in their healthcare organizations, some flagged that DOACs errors were still occurring:

“………so I think we have an excellent system however we still have significant DOACs medication error that I am not happy about” Participant 8, physician (Riyadh)

There were differences in participant perceptions around quality and adequacy of safety policies depending on the regions participants belonged to.

“Yes, we don’t have a clear medication safety policy, which is not clear used by all our physicians, and those who are concerned with treatment in, in cases of thrombosis from particulates.” Participant 4, physician (Jazan)

Participants described that electronic prescribing systems can promote safe use of DOACs:

“So, essentially, we have a very helpful electronic healthcare system in organization and all medications are ordered through this system, so actually within this system, there is an extremely sensitive drug interaction, alert system that picks up any interaction, so in this system is exceptionally helpful in monitoring these interactions.” Participant 9, physician (Riyadh)

However, the unavailability of several DOACs choices of medications within the hospital caused some frustration and challenges for some participants. Having only one choice would put the treating physician in a situation to hesitate to prescribe.

“Okay, availability of the medications. So, sometimes, I feel this anticoagulant is better than this so, for example, I feel Apixaban is better than, for example, Dabigatran but, it's not available. So, the availability also of the medication.” Participant 1, physician (Jazan)

### Factors related to DOACs

Participants indicated that lack of idarucizumab availability in Saudi Arabia to counteract the risk of bleeding that may result from DOACs can affect patients’ safety while receiving these medications. The non-availability of idarucizumab could make physicians prescribe under-dosing anticoagulation.

“If the patient unfortunately has bleeding, there is no antidote for direct oral anticoagulant, that's the main concern for us if the patient has a bleeding so, the bleeding is the main issue for us as physician.” Participant 1, physician (Riyadh)

Participants stressed concerns regarding the difficulty that they face in monitoring these agents due to their lack of experience in their use as compared to warfarin:

“…monitoring of anticoagulant affects these agents is problematic … in comparison to warfarin which is essentially universal.” Participant 9, physician (Riyadh)

Workload was deemed to impact on DOACs safety. In particular, many participants described having seen their workload increase as a result of the emergence of COVID-19 pandemic:

“…one of it is the workload factor because we are working in COVID-19 area, so the thing is that most of the nurses, can be handling 3 patients, the workload itself is sometimes (a problem) …” Participant 9, Nurse (Riyadh)

COVID-19 was deemed to have impacted patients' response to DOACs efficacy:

“…if we have a patient with corona (COVID-19), and for discharge, we, we keep him on anticoagulant, apixaban, for two weeks at home, as a prophylaxis only. This is not actually, this is not international guideline but, this is local guidelines based on our observation. We have, we have seen many cases, we discharge them, and they come back within like five, ten days with deep venous thrombosis (DVT) or pulmonary embolism (PE).” Participant 1, physician (Jazan)

### Suggested strategies for prevention/mitigation of errors

Participants provided several suggestions to help minimize errors related to the use of DOACs.

Educational initiatives and training opportunities could enhance their understanding and prescribing practices of DOACs:

“… I think we need more education and lectures,.. we are developed …. our institute is one of the leading institutes that come into medication safety in particular, but as I said are we perfect? No, we are not” Participant 4, physician (Riyadh)

A few highlighted the positive impact of the healthcare accreditation process on the safe use of medications in general and high-risk medications including the DOACs. Most physicians clearly stated the role of medications by joint commission international (JCI) and the Saudi Central Board for Accreditation of Healthcare Institutions (CBAHI) which is the official agency authorized to grant accreditation certificates to all healthcare facilities operating in Saudi Arabia. This accreditation process even includes the pharmacy and high alert medications, their prescription, dispensing and counselling associated with the dispensing process in order to improve their effectiveness and safety.

“We have JCI [joint commission international] and CBAHI [the Saudi Central Board for Accreditation of Healthcare Institutions] so, regarding the pharmacy, regarding the prescription, electronic prescription, this is, will minimize the DOACs medication errors really, even if you, the system by default, if you write apixaban, by default it will come twice daily(BID)” Participant 6, physician (Dhahran)

HCPs stated that paying careful attention to the baseline assessment of patients’ risk of bleeding prior prescribing DOACs was a helpful strategy to avoid accidental bleeding from these agent as described by this physician:

“I'm using, a score to check the bleeding risk for my patient. I have application on my smart phone and then I check the risk of bleeding and I classify my patient, if my patient at high risk of bleeding, I discuss with the patient, or patient relative and, I, you know, I make a balance between if it is strongly indicated and then we make a shared decision between me and a patient” Participant 1, physician (Jazan)

Another physician described the importance of carefully balancing the risk benefit profile prior to DOACs initiation would promote safety and minimize errors:

“first of all you have to review the indication, before starting the prescribing or even thinking about it, what is the indication ok? What is the risk and benefit and then taking the patient’s factors like patient preference, patient risk factors, patient comorbidities?” Participant 6, physician (Jazan)

Most participants agreed on the need for regular monitoring of the patients receiving DOACs to ensure the efficacy and safety of these medications.

Participants stressed on the need for adequate patient education and implementation of the medication reconciliation prior to patient’s discharge from hospital to support the safe use of medications:

“We usually before discharge the patient with this agent, we sit with them and explain very clearly to the patients, when to stop this and when to stop that and when you come to us again, we explained that.” Participant 7, physician (Riyadh)

HCPs reported that confidence in prescribing and administering DOACs in not harming the patients relied on proper patient education:

“.. I'm confident to administer this medication to our patient, as long as they will be educated on…” Participant 8, Nurse (Riyadh)

## Discussion

### Statement of key findings

This study explored the perception of HCPs about DOACs medication errors. A range of factors related to HCPs, patients, and healthcare organizations and opportunities were identified.

Many participants described that they did not have sufficient knowledge about DOACs. Lack of local protocols, clinical guidelines, information resource were described. Implementation of an educational intervention approach that provides HCPs with the knowledge and training about DOACs and encouraging a discussion about the DOACs medication errors might help to improve safe use of DOACs [16]. Furthermore, our study showed a lack of participant access to national and/or instititional guidelines on the use of DOACs.

Participants in this study emphasised that effective interprofessional collaboration and effective communication among HCPs can play a huge role in promoting DOACs safety. Previous evidence refers to effective communication and teamwork as core elements for providing safe care [17–19]. Communication failures are an extremely common cause of inadvertent patient harm [19]. Using structured communication tools through digital applications and multidisciplinary team meetings can be effective such as using teleconferences and the digital Walkie-talkie mobile [20].

### Interpretation

Our results showed that participants’ confidence in prescribing DOACs to patients were boosted if they collaoborated with clinical pharmacists. Studies have shown that involvement of pharmacists in the appropriate selection of DOACs doses decreased errors [21, 22]. Additionaly, pharmacists were able to reduce DOACs errors through medications reviews conducted during the hospital stay, at the clinics and through medication reconciliation [21, 22].

Patient-centered care was identified to be important in reducing medication errors. Physicians reported that patient and family involvement in a shared decision-making process when prescribing DOACs (especially if replacing warfarin for DOACs) was crucial. Studies have shown that patient engagement and adherence to treatment are essential to acheiving optimal treatment outcomes with DOACs [23].

Participants of this study revealed factors associated with DOACs errors such as patients age, health condition, socio-economic status, and polypharmacy. Studies have shown that physicians might underdose and/or misdose patients who are elderly or taking a lot of medications [24–27]. HCPs require guidance when managing complex patients as most clinical guidelines focus on single disorders.

The participants of this study also highlighted the importance of safety culture in healthcare orgnanisations. Medication safety programmes have been introduced in many hospitals to minimize the likelihood of harm associated with the use of medication, and maximize the benefits for patients [28]. Adopting facets like medication safety policy, multidisciplinary team policy, double checking policy as well as adopting electronic prescribing system are common safety practices activated in hospitals [29].

Hospital accreditation standards were deemed effective to improve the safety culture and minimise errors. Since 2006, the ministry of health represented by Saudi Central Board for Accreditation of Healthcare Institutions (CBAHI) which is the official agency authorized to grant accreditation certificates to all governmental and private healthcare facilities operating in Saudi Arabia, required an accreditation certification for central hospitals across Saudi Arabia. In addition, the MOH adopted other perfomrance monitoring systems and quality improvement programs known as “Adaa” and “Wazen” programs to assess hospital performance agaist an explicit set of standards that included 30 essential indicators focusing on safety and quality improvement of clinical practice in hospitals. The ministry executed vocational and educational programmes to spread the safety culture among HCPs through training. Furthermore, the MOH improved the functions of pharmacy and therapuctice committee, establihsed new adminstration for drug regulation and control, and adopted formal arrangement between pharmaceutical companies and payers or regulators to improve access to costly, innovative medicines. As a result, idarucizumab recently become available in Saudi Arabia[30].

### Strengths and weaknesses

Involvment of participants from different regions with diverse patient populations, variable healthcare practice enhances the transferability of the findings to other regions in Saudi Arabia. In addition, many of the factors identified including organisational, patient and prescriber related factors are likely to be applicable internationally as evidenced by other publications described in the discussion section. However, differences in organizational and quality of health care such as staffing levels, workload and availability of advanced electronic prescribing systems can impact the transferability of findings to other settings within Saudi Arabia and beyond and this warrants further explorations of the same issue inother regions.

### Further research

Qualitative studies consisting of semi-structured interviews of patients and pharmacists to further explore causes and ways to minimise DOACs medication incidents are needed. Additional research should aim to extend the scope of this study to incident severity and its impact on patient health outcomes. Development and evaluations of interventions to minimise errors are needed. Research should be extended to non-hospital settings.

## Conclusion

Our study highlighted important contributory factors to medication errors related to DOACs from the perspective of HCPs. A gap in knowledge and training when dealing with DOACs requires standard guidelines and educational initiatives to support optimal utilization of DOACs. Furthermore, attention should be drawn to the prescribing process being a vital step that requires multi-disciplinary collaboration. Involvement of pharmacists at the prescribing, monitoring and follow-up was identified as a key strategy that can increase physicians’ and nurses’ confidence and minimize errors.

## Supplementary Information

Below is the link to the electronic supplementary material.

Supplementary file1 (DOCX 19 KB)

## References

[1] Al-Hameed FM, Al-Dorzi HM, Al-Momen AM, Algahtani FH, Al-Zahrani HA, Al-Saleh KA (2015). The Saudi Clinical Practice Guideline for the treatment of venous thromboembolism. Outpatient versus inpatient management. Saudi Med J..

[2] Lopez Valle RG (2014). Summary of evidence-based guideline update: prevention of stroke in nonvalvular atrial fibrillation: report of the guideline development subcommittee of the American academy of neurology. Neurology.

[3] Ezekowitz MD, Connolly S, Parekh A, Reilly PA, Varrone J, Wang S (2009). Rationale and design of RE-LY: randomized evaluation of long-term anticoagulant therapy, warfarin, compared with dabigatran. Am Heart J.

[4] Heidbuchel H, Verhamme P, Alings M, Antz M, Diener H-C, Hacke W (2015). Updated European Heart Rhythm Association practical guide on the use of non-vitamin K antagonist anticoagulants in patients with non-valvular atrial fibrillation. EP Europace.

[5] Lippi G, Mattiuzzi C, Cervellin G, Favaloro EJ (2017). Direct oral anticoagulants: analysis of worldwide use and popularity using Google Trends. Ann Transl Med.

[6] Wu Y, Zhang C, Gu ZC (2021). Cost-effectiveness analysis of direct oral anticoagulants Vs. Vitamin k antagonists in the elderly with atrial fibrillation: insights from the evidence in a real-world setting. Front Cardiovasc Med..

[7] Xu R, Wu F, Lan J, Duan P (2021). Real-world comparison of direct-acting oral anticoagulants and vitamin K antagonists in chronic kidney disease: a systematic review and meta-analysis. Expert Rev Hematol.

[8] Navarro-Almenzar B, Cerezo-Manchado JJ, Garcia-Candel F (2021). Real life behaviour of direct oral anticoagulants in patients with nonvalvular atrial fibrillation and morbid obesity. Int J Cardiol Heart Vasc.

[9] Al Rowily A, Jalal Z, Price MJ, et al. Prevalence, contributory factors and severity of medication errors associated with direct-acting oral anticoagulants in adult patients: a systematic review and meta-analysis. Eur J Clin Pharmacol. 2021:1–23. 10.1007/s00228-021-03212-y PMC8926953 34935068

[10] Michie S, Van Stralen MM, West R (2011). The behaviour change wheel: a new method for characterising and designing behaviour change interventions. Implement Sci.

[11] Alrowily A, Jalal Z, Abutaleb MH, Osman NA, Alammari M, Paudyal V (2021). Medication errors associated with direct-acting oral anticoagulants: analysis of data from national pharmacovigilance and local incidents reporting databases. J Pharm Policy Pract.

[12] Viprey M, Jeannin R, Piriou V, Chevalier P, Michel C, Aulagner G (2017). Prevalence of drug-related problems associated with direct oral anticoagulants in hospitalized patients: a multicenter, cross-sectional study. J Clin Pharm Ther.

[13] Haque H, Alrowily A, Jalal Z, Tailor B, Efue V, Sarwar A (2021). Direct oral anticoagulant-related medication incidents and pharmacists’ interventions in hospital in-patients: evaluation using reason’s accident causation theory. Int J Clin Pharm.

[14] Reason J (1995). A systems approach to organizational error. Ergonomics.

[15] Kuckartz U, Rädiker S (2019). Analyzing qualitative data with MAXQDA.

[16] Piran S, Schulman S, Panju M, Pai M (2018). Oral anticoagulant dosing, administration, and storage: a cross-sectional survey of Canadian health care providers. J Thromb Thrombol.

[17] O'Daniel M, Rosenstein AH. Professional communication and team collaboration. In: Hughes RG, editor. Patient safety and quality: an evidence-based handbook for nurses. Advances in Patient Safety. Rockville (MD)2008.

[18] Sennesael A-L, Larock A-S, Devalet B, Mathieux V, Verschuren F, Muschart X (2018). Preventability of serious thromboembolic and bleeding events related to the use of oral anticoagulants: a prospective study. Br J Clin Pharmacol.

[19] Leonard M, Graham S, Bonacum D (2004). The human factor: the critical importance of effective teamwork and communication in providing safe care. Qual Saf Health Care..

[20] Pokrovskaia NN, Leontyeva VL, Ababkova MY, Cappelli L, D’Ascenzo F (2021). Digital communication tools and knowledge creation processes for enriched intellectual outcome—experience of short-term E-learning courses during pandemic. Fut Internet.

[21] Willeford A, Leiman V, Noel ZR. Impact of a pharmacist‐to‐dose direct oral anticoagulant protocol on medication errors at an academic medical center. J Am Coll Clin Pharm. 2021;4(11):1392–400.

[22] Musy E, Hiel C, Poutrain E, et al. Role of a clinical pharmacist in cardiology units: Let's take the example of medication reconciliation (MR)! Arch Cardiovas Dis Suppl. 2021;13:151.

[23] Moudallel S, van den Bemt BJ, Zwikker H, de Veer A, Rydant S, van Dijk L (2021). Association of conflicting information from healthcare providers and poor shared decision making with suboptimal adherence in direct oral anticoagulant treatment: a cross-sectional study in patients with atrial fibrillation. Patient Educ Couns.

[24] Cavallari I, Patti G (2018). Efficacy and safety of oral anticoagulation in elderly patients with atrial fibrillation. Anatol J Cardiol.

[25] Abohelaika S, Wynne H, Avery P, Robinson B, Kesteven P, Kamali F (2016). Impact of age on long-term anticoagulation and how gender and monitoring setting affect it: implications for decision making and patient management. Br J Clin Pharmacol.

[26] Fernandez CS, Gullon A, Formiga F (2020). The problem of underdosing with direct-acting oral anticoagulants in elderly patients with nonvalvular atrial fibrillation. J Comp Eff Res.

[27] Bellia A, Della-Morte D, Di Daniele N, Lauro D (2021). Drug interactions of direct oral anticoagulants in elderly patients with cardiometabolic diseases. Curr Res Pharmacol Drug Discovery.

[28] Bennie M, Kurdi A, MacBride-Stewart S, Avery T (2021). Medication safety in primary care—from measurement to action. Drug Ther Bull.

[29] Dilles T, Heczkova J, Tziaferi S, Helgesen AK, Grøndahl VA, Van Rompaey B (2021). Nurses and pharmaceutical care: interprofessional, evidence-based working to improve patient care and outcomes. Int J Environ Res Public Health.

[30] Managed Entry Agreement Policy for Saudi MOH [Drug Policy and Regulation.]. https://www.moh.gov.sa/en/Ministry/MediaCenter/Publications/Pages/MOH-Drug-policy-and-regulation-.aspx. Accessed 08 June 2022.

