# Supplementary material for: Views, experiences and contributory factors related to medication errors associated with direct oral anticoagulants: a qualitative study with physicians and nurses
Source: Int J Clin Pharm. 2022 Jun 22;44(4):1057–66. doi: 10.1007/s11096-022-01448-x (PMC9393124; doi:10.1007/s11096-022-01448-x)
Supplement: Supplementary file 1 — Supplementary file1 (DOCX 19 KB) [file 11096_2022_1448_MOESM1_ESM.docx]

# Electronic supplement 1

**Qualitative interview schedule**

1. What is your experience in dealing with DOAC ( prescribing, dispensing (processing), or administration)
   - Prompts (This might include, number of years, being involved in formulary selection, mortality& morbidity committee, patients counselling, educational campaign..
2. What are the key concerns with the use of DOACs from your perspective at your institution?
3. How knowledgeable are you in terms of prescribing/dispensing/administring DOACs?
   - Prompting (what about your colleagues, the doses and frequency, when to stop before procedures, side effects, drug pharmacokinetics, indications, and contraindications, safety in pregnancy and lactation, possible drug and food interactions ( ask for examples)
4. Do you think that medication use process (MUP) (add more simpler terms about the process as prompting words)is safe at your institution? The perceived safety culture of the hospital).
   - In general, from your prospective how safe is prescribing, dispensing and administration practice of medicines at your institution? Prompting (Do you have medication safety policy, or DOACs guidelines at your institution? Are you familiar with it?
5. Do you have guidelines to follow? What guidelines are you following when you deal with DOAC in adult population with different clinical conditions
6. Does these guidelines address your needs to deal with DOAC patients?
   - Answer this question if the previous question is answered
7. What other information sources (in addition to the guidelines) you use when you deal with DOACs?
8. How skilful do you feel about prescribing/dis/admins of DOACs?
9. Have you had any training or Continuing Medical Education (CME) opportunities to ensure safe practice of DOACs? And if yes, what are they?
   - How common are medication errors associated with DOACs?
   - Have you been involved in medication errors, related to DOACs? If yes, please describe prompting (What was the medication error type? how old was the patient? happened during the shift? was the patient admitted to the hospital or the length of stay prolonged? does the patient required medical intervention to reverse the harm?

From your perspective in this error, what was the contributory factors at that incidence?

- - Contributory factors (What are the potential factors from the error theories?
  - What could have been done differently to minimise the errors?

1. What can be done to minimise errors associated with these medicines, generally speaking?
2. What preventive safety measures should be implemented to minimize medication error associated with DOAC before prescribing, at your institution? How often are patients assessed for their needs based on their clinical circumstances in your setting before being prescribed DOACs?
3. How often are patients assessed for risks of bleeding based on their clinical circumstances in your setting before being prescribed DOACs?
4. How motivated do you think to assess risks in patients?
5. Whose role is it to monitor patients for risks of bleeding and other adverse events in relation to DOACs?
6. Do you think that the current policies and procedures or guidelines at your institution are efficient to maintain patient safety when using DOAC? If no why and what barriers are existing to hender applying these policies by HCW

**Overal**l, what do you think are the most important strategies for preventing prescribing, dispensing, and administration errors in the current practice?

Additional prompts for the researcher to discuss contributory factors

- - **Patient characteristics** [prompt: what factor relating to the patient contribute to errors such as seriousness and complexity of their conditions, communication challenges or social factors.]
  - **Knowledge of the patient** [prompt: does familiarity with patient make errors more or less likely?]
  - **Your therapeutic knowledge and prescribing, dispensing, administer skills**[prompt: how familiar are you with drug, how do you feel about your knowledge of therapeutics and skills]
  - **Your information sources and technology** [prompt: what information do you use to help guide you and how useful is this in preventing error?]
  - **Task factors** [prompt: is there anything about the way in which prescribing, dispensing, administer task are organised and structured in your practice that might lead to increased risk of errors. E.g: available information on the patient and drug at the point of decision making, competing priorities for time during consultation, interruptions, lack protocol for safe prescribing, dispensing, administer including repeat prescribing and refill]
  - **Your working environment** [prompt: what do you think of the working environment within your working day? To what extent(and how) does it contribute to the occurrences of (or prevention of) prescribing, dispensing and administer errors like possible factors including staffing levels and skills mix, workload and shift pattern, design, availability and maintenance of equipment, administrative and managerial support]
  - **Team factors** [prompt: what do you think of the team factors within your working day? To what extent do teamwork, team structures and communication contribute to the prescribing, dispensing and administer drug errors, or prevent them for happening? Like possible related factors include verbal communication, written communication, supervision and seeking help]
  - **Organisation and management factors** [prompt: to what extent do you think the organisation and management within the practice contribute to prescribing, dispensing and administer or preventing them? What do you think of the safety culture within the practice like possible related factors including financial resources and constraints, organisational structure, policy standards, goals and priorities ]
  - **Individual factors** [prompt: are there any personal strategies you have for helping to prevent medication errors? Conversely, do you feel there are sometimes individual factors that put you at increased risk of making errors?]
  - **Are there any other factors** that you think may important in preventing prescribing, dispensing and administer errors with DOACs? [Prompt: can you describe the factors that you feel interfere with your ability to safely prescribe, dispense and administer drug error?
  - **Imagine that you have to teach a doctors, pharmacists and nurse** how to safely administer DOACs. What information would you consider essential for the doctors, pharmacists and nurse to know for preventing errors?

**Closing:**

1. Is there anything else you did not tell me yet but think may be of interest?
2. Is there anything that you would like to go back and talk about?
3. Switch off tape recorder. Thank the participants.
